# Inactivation of the TIM complex components leads to a decrease in the level of DNA import into Arabidopsis mitochondria

**DOI:** 10.18699/VJGB-23-112

**Published:** 2023-12

**Authors:** T.A. Tarasenko, K.D. Elizova, V.I. Tarasenko, M.V. Koulintchenko, Yu.M. Konstantinov

**Affiliations:** Siberian Institute of Plant Physiology and Biochemistry of the Siberian Branch of the Russian Academy of Sciences, Irkutsk, Russia; Siberian Institute of Plant Physiology and Biochemistry of the Siberian Branch of the Russian Academy of Sciences, Irkutsk, Russia; Siberian Institute of Plant Physiology and Biochemistry of the Siberian Branch of the Russian Academy of Sciences, Irkutsk, Russia; Siberian Institute of Plant Physiology and Biochemistry of the Siberian Branch of the Russian Academy of Sciences, Irkutsk, Russia Kazan Institute of Biochemistry and Biophysics of Kazan Scientific Center of the Russian Academy of Sciences, Kazan, Russia; Siberian Institute of Plant Physiology and Biochemistry of the Siberian Branch of the Russian Academy of Sciences, Irkutsk, Russia

**Keywords:** mitochondria, DNA import, Tim17, Tim23, VDAC1, transport channel, knock-out mutant, Arabidopsis thaliana, митохондрии, импорт ДНК, Tim17, Tim23, VDAC1, транспортный канал, нокаут-мутант, Arabidopsis thaliana

## Abstract

The phenomenon of DNA import into mitochondria has been shown for all major groups of eukaryotes. In plants and animals, DNA import seems to occur in different ways. It has been known that nucleic acids enter plant organelles through alternative channels, depending on the size of the imported molecules. Mitochondrial import of small DNA (up to 300 bp) partially overlaps with the mechanism of tRNA import, at least at the level of the outer membrane. It is noteworthy that, in plants, tRNA import involves components of the protein import apparatus, whose role in DNA transport has not yet been studied. In this work, we studied the role of individual components of the TIM inner membrane translocase in the process of DNA import into isolated Arabidopsis mitochondria and their possible association with the porin VDAC1. Using knockout mutants for the genes encoding Tim17 or Tim23 protein isoforms, we demonstrated for the first time the involvement of these proteins in the import of DNA fragments of different lengths. In addition, inhibition of transport channels with specific antibodies to VDAC1 led to a decrease in the level of DNA import into wild-type mitochondria, which made it possible to establish the specific involvement of this porin isoform in DNA import. In the tim17-1 knockout mutant, there was an additional decrease in the efficiency of DNA import in the presence of antibodies to VDAC1 compared to the wild type line. The results obtained indicate the involvement of the Tim17-1 and Tim23-2 proteins in the mechanism of DNA import into plant mitochondria. At the same time, Tim23-2 may be part of the channel formed with the participation of VDAC1, while Tim17-1, apparently, is involved in an alternative DNA import pathway independent of VDAC1. The identification of membrane carrier proteins involved in various DNA import pathways will make it possible to use the natural ability of mitochondria to import DNA as a convenient biotechnological tool for transforming the mitochondrial genome.

## Abstract (ru)

Феномен импорта ДНК в митохондрии показан для всех основных групп эукариот. В растениях и животных импорт ДНК, по-видимому, происходит различными путями. Известно, что в растительные органеллы нуклеиновые кислоты попадают по альтернативным каналам в зависимости от размера импортируемых молекул. Импорт ДНК небольшого размера (до 300 п. н.) частично перекрывается с механизмом импорта тРНК, по крайней мере, на уровне внешней мембраны. Примечательно, что у растений в импорт тРНК вовлечены компоненты аппарата импорта белков, чья роль в транспорте ДНК до настоящего времени оставалась неизученной. В настоящей работе мы провели исследование роли отдельных компонентов транслоказы внутренней мембраны TIM в процессе импорта ДНК в изолированные митохондрии арабидопсиса и их возможной связи с пори- ном VDAC1. С использованием нокаут-мутантов по генам, кодирующим изоформы белков Tim17 или Tim23, мы впервые показали участие этих белков в импорте фрагментов ДНК разной длины. Кроме того, ингибирование транспортных каналов специфическими антителами к VDAC1 приводило к снижению уровня импорта ДНК в митохондрии дикого типа, что позволило установить специфическое участие этой изоформы порина в импорте ДНК. В нокаут-мутанте tim17-1 происходило дополнительное снижение эффективности импорта ДНК в присутствии антител к VDAC1 в сравнении с линией дикого типа. Полученные результаты указывают на участие белков Tim17-1 и Tim23-2 в аппарате импорта ДНК в растительные митохондрии. При этом Tim23-2 может быть частью канала, формируемого при участии VDAC1, в то время как Tim17-1, по-видимому, вовлечен в альтернативный, независимый от VDAC1, путь импорта ДНК. Выявление мембранных белков-переносчиков, участвующих в различных путях импорта ДНК, позволит использовать природную способность митохондрий к поглощению ДНК в качестве удобного биотехнологического инструмента для трансформации митохондриального генома.

## Introduction

Mitochondria are double-membrane organelles of aerobic eukaryotes that are responsible for providing energy to the cell and have their own genetic system. Mitochondrial DNA (mtDNA), a legacy of the endosymbiotic event (Martin et al., 2015), encodes rRNA, tRNA, ribosomal proteins, and oxidative phosphorylation proteins (Morley, Nielsen, 2017). The ability to modify the mitochondrial genome can become a convenient tool for making targeted changes in mtDNA in order to obtain plants with valuable agricultural characteristics and solve issues of gene therapy treatment of human mitochondrial diseases.

The methodology for transforming mitochondria with exogenous DNA is at the initial stages of its development (Larosa, Remacle, 2013), since effective methods for manipulating the mitochondrial genome using targeted delivery of nucleic acid molecules have not yet been established. One promising approach to transforming the mitochondrial genome could be manipulation of the DNA import process – the natural ability of mitochondria to uptake DNA from the cytoplasm

The phenomenon of DNA import was initially demonstrated for plants (Koulintchenko et al., 2003; Konstantinov et al., 2016), but was subsequently described for mammalian mitochondria (Koulintchenko et al., 2006) and yeast (Weber- Lotfi et al., 2009). It should be noted that currently there is no complete understanding of how the transmembrane transfer of DNA into the mitochondrial matrix occurs. Apparently, DNA import involves different pathways in plants and mammals (Koulintchenko et al., 2006). Moreover, in plant mitochondria, DNA transfer can occur through several alternative mechanisms involving a variety of protein complexes (Weber-Lotfi et al., 2015; Tarasenko et al., 2021).

DNA import into plant mitochondria throughout the outer membrane occurs with the participation of porin (VDAC, voltage- dependent anion channel) (Koulintchenko et al., 2003). The role of VDAC has also been shown in tRNA import (Salinas- Giegé et al., 2015), a cellular process that ensures the functioning of the genetic system of these organelles (Morley, Nielsen, 2017). Several VDAC isoforms are present in plant cells (Tateda et al., 2011); in particular, there are four functional isoforms in Arabidopsis thaliana (Tateda et al., 2011) that perform different roles. VDAC1 is more important for plant growth and disease resistance (Tateda et al., 2011), while VDAC3 appears to be involved in the stress response (Hemono et al., 2020). Based on the tRNA binding intensity of four VDAC isoforms, it was suggested that VDAC4 is involved in the import of tRNA into mitochondria (Hemono et al., 2020).

Differential interaction of mitochondrial porins with tRNA is also characteristic of other plants. Thus, only VDAC34 appears to be involved in tRNA import in potato, since this isoform shows strong binding to the tRNA molecule (Salinas et al., 2014). Arabidopsis VDAC isoforms could potentially also specialize in DNA transport depending on the length of the imported molecules (Tarasenko et al., 2021). In Arabidopsis knockout lines lacking VDAC1, VDAC2, or VDAC4, there was an increase in DNA import accompanied by induction of VDAC3 expression, which could be part of a cellular mechanism aimed at compensating for the absence of a porin isoform.

Import of small DNA (up to 300 bp) through the inner mitochondrial membrane may occur with the participation of adenine nucleotide transporters (ADNT1), ATP-Mg/Pi (APC) (Tarasenko et al., 2021) and/or phosphate transporter MPT (Weber-Lotfi et al., 2015). The import of medium-sized DNA (400–7000 bp) involves the AAC adenine nucleotide transporter (ADP/ATP carrier) (Koulintchenko et al., 2003). The involvement of CuBP, a subunit of respiratory complex I, in the transport of medium- and large-sized DNA (Weber-Lotfi et al., 2015) appears to be related to the stabilization of the channel through which larger molecules are transported.

The competitive inhibition method has been used to demonstrate the possible interplay of the import pathways of tRNA and small DNA (Weber-Lotfi et al., 2015), which is not surprising given the involvement of VDAC in the import of both tRNA (Salinas et al., 2006) and DNA (Koulintchenko et al., 2003; Tarasenko et al., 2021). It is noteworthy that components of the protein import apparatus are also involved in the process of tRNA import in plants (Verechshagina et al., 2018). This fact indicates the multifunctionality of some membrane transporters in plant mitochondria. Based on these data, it is logical to assume that the components of protein complexes involved in the processes of tRNA and/or protein translocation occurring in plant mitochondria may also be involved in the DNA import mechanism.

The most obvious candidate for the role of a multifunctional transporter appears to be the TIM complex, also known as TIM17:23, which is responsible for the transport of proteins into the mitochondrial matrix. This membrane complex is directly linked into a single channel with the translocase of outer mitochondrial membrane proteins TOM (translocase of the outer membrane), individual components of which are involved in tRNA import in plants (Salinas et al., 2006).

The inner membrane translocase TIM17:23, one of the largest mitochondrial protein complexes, consists of two main subunits – Tim17 and Tim23. This protein is anchored in the inner membrane by four transmembrane helices, forming a translocation channel (Ryan et al., 1998; Truscott at al., 2001). It is known that the Tim23 subunit is responsible for the formation of the pore, and Tim17 is responsible for the stabilization and regulation of this pore (Verechshagina et al., 2018). Each of these subunits has three isoforms in Arabidopsis plants (Murcha et al., 2007), but the degree of their participation in protein import appears to be different, indicating their potential role in other cellular processes.

It is known that the TIM17:23 complex is dominated by the Tim23-2 subunit, which is characterized by the highest level of expression in all tissues (Murcha et al., 2003). Considering the high degree of homology of Tim23 isoforms, indicating their potentially interchangeable properties, it cannot be excluded that the predominant Tim23-2 isoform may have a multifunctional role in cellular processes, similar to what was established for the main components of the TOM complex (Salinas-Giegé et al., 2015). Notably, the Tim23-2 subunit is present in respiratory complex I in addition to TIM17:23 (Murcha et al., 2005; Wang et al., 2012). The Tim23-3 subunit, in contrast, has a low level of expression and is the most divergent in sequence from the other two isoforms (Murcha et al., 2007), which may indicate that this protein performs additional functions.

The most common Tim17 isoform in Arabidopsis is the Tim17-2 protein, which is characterized by a consistently high level of expression throughout development. The Tim17- 1 isoform has a fairly high (75 %) degree of similarity to Tim17- 2 (Wang et al., 2014). It should be noted that to date there are no unambiguous data on the role of Tim17-1 in plants (Wang et al., 2014). Unlike Tim17-2, the Tim17-1 isoform is characterized by changes in expression levels depending on the developmental stage, with the most pronounced increase at the seed development stage, but a gradual decrease with development (Wang et al., 2014). Obviously, protein import is ensured by the predominant isoforms in an adult plant, while the minor Tim17-1 could potentially specialize in performing functions not related to mitochondrial biogenesis

In our work, we showed that the Tim23-2 and Tim17-1 proteins are involved in the transmembrane transfer of DNA into the mitochondrial matrix, while Tim23-2 appears to be responsible for the import of exclusively short fragments. In addition, the VDAC1 porin isoform appears to be directly involved in the process of DNA import and is likely part of a channel formed with the participation of the Tim23-2 protein. The data obtained open up prospects for further studies of the role of TIM components in the import of nucleic acids into mitochondria.

## Materials and methods

Plant material and growing conditions. We used wild- type Arabidopsis thaliana (L.) Heynh. ecotype Columbia (Col-0) plants and GABI_689C11 (tim23-2, At1g72750 gene), SALK_ 129386 (tim23-3, At3g04800 gene) and SALK_092885 (tim17-1, At1g20350 gene) knockout lines. Seeds of these lines were provided by Monika Murcha (ARC Center of Excellence in Plant Energy Biology, Perth, Australia). Seeds were subjected to stratification for 3 days at 4 °C, and then grown at 22 °C in a KBW720 growth chamber (Binder, Germany) in pots filled with a compost/vermiculite mixture in a ratio of 2:1 at a photosynthetic photon flux density of 150 μmol m–2 · s–1 and 16-hour photoperiod.

Preparation of DNA import substrates. For DNA amplification, Taq polymerase (Thermo Scientific, USA) was used in accordance with the manufacturer’s recommendations. The genetic construct pCK/GFP/PRmt (Koulintchenko et al., 2003), containing the GFP gene sequence, was used as a PCR template.

Amplification of DNA fragments of 2732 bp (Forward: 5ʹ-CCAACCACCACATACCGAAA-3ʹ; Reverse: 5ʹ-ACGCT CTGTAGGATTTGAACC-3ʹ) and 265 bp (Forward: 5ʹ-AT GAGTAAAGGAGAAGAACTTTTCACT-3ʹ; Reverse: 5ʹ-CGGGGCATGGCACTCTTGA-3ʹ) containing the GFP gene sequence was carried out using specific primer pairs at an annealing temperature of 60 °C. DNA was purified using GeneJET™PCR Purification Kit columns (Thermo Scientific) according to the manufacturer’s instructions. The quality of PCR products was assessed electrophoretically using the Gel Doc XR System (Bio-Rad, USA), the amount of DNA was determined using a NanoPhotometer NP80 spectrophotometer (IMPLEN, Germany).

Isolation of mitochondria. A crude mitochondrial extract was prepared from 3-week-old A. thaliana plants according to a previously described protocol (Sweetlove et al., 2007) by differential centrifugation. The purified mitochondrial fraction was obtained by separating the crude mitochondrial fraction in a stepwise Percoll density gradient (50–28–20 %) for 40 min at 40,000 g. A suspension of mitochondria was collected at the boundary of layers with 50 and 28 % Percoll concentrations.

Import of DNA substrates into Arabidopsis mitochondria in organello. DNA import was performed as described previously (Tarasenko et al., 2019). 200 μg of purified mitochondria were added to 200 μl of import buffer (0.4 M sucrose, 40 mM potassium phosphate, pH 7.0) containing 500 ng of DNA, then incubated at 25 °C for 30 min. Mitochondria were treated with DNase I (1 unit/μl) (Thermo Scientific) in 100 μl of import buffer in the presence of 10 mM MgCl2 for 20 min at 25 °C. Samples were then washed in wash medium containing additional 10 mM EDTA and 10 mM EGTA, and mtDNA was extracted for further analysis of DNA import efficiency. As a control for the efficiency of DNase treatment, a sample without the addition of mitochondria was used. The level of the background signal obtained from such a sample was always at least two orders of magnitude lower than the level of the signal from import samples.

Preparation of protoplasts, transfection with DNA molecules and isolation of mitochondria. Protoplasts were obtained from A. thaliana leaves according to a previously described protocol (Wu et al., 2009) with modifications (Tarasenko et al., 2019). A DNA substrate (5 μg) was added to the suspension of isolated protoplasts, after which 300 μl of a solution containing 20 % PEG-2000, 0.2 M mannitol, 100 mM CaCl2 was added to the samples. The protoplasts were incubated for 5 min, then the protoplast suspension was subjected to three cycles of centrifugation in 1.5 ml of washing medium for 1 min at 100 g and 20 °C. Further incubation of the protoplasts was carried out in W5 medium (154 mM NaCl, 125 mM CaCl2, 5 mM KCl, 5 mM glucose, 2 mM MES, pH 5.7) at 22 °C and low light for 20 hours. The protoplast suspension was centrifuged for 1 min at 100 g and 20 °C and mitochondria were isolated as described previously (Tarasenko et al., 2019).

DNA import assay. The amount of DNA imported into mitochondria was determined by quantitative PCR (qPCR) using the qPCRmix-HS SYBR kit (Evrogen, Russia) according to the manufacturer’s instructions. Data were analyzed using CFX Manager software (Bio-Rad). We used primer pairs specific for the GFP gene sequence (Forward: 5ʹ-GAT GTGGAAAACAAGACAGGGGTTT-3ʹ; Reverse: 5ʹ-TGG TGAACCGGGCGTACTATTT-3ʹ) and the NAD4 gene sequence from the Arabidopsis mitochondrial genome (Forward: 5ʹ-GCATTTCAGTGGGTTGGTCTGGT-3ʹ; Reverse: 5ʹ-AGGGATTGGCACGCTTTCGG-3ʹ). The ratio of the content of imported DNA to mtDNA was calculated based on the ratio of the absolute values of the signal from the imported DNA and from the NAD4 gene, taking into account the difference between the sizes of the imported fragments (265 bp and 2.7 kb) and the mt genome (367 kb) with the assumption that the mt genome is represented exclusively by the master chromosom

Statistical analysis. Experiments were carried out in at least three biological replicates. The diagrams were constructed using the Microsoft Excel software package. The degree of significance of differences was assessed using Student’s test.

## Results

**Fig. 1.**
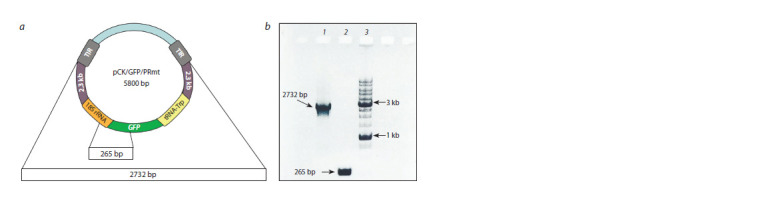
DNA substrates used for import into Arabidopsis mitochondria. а – scheme of the pCK/GFP/PRmt genetic construct (Koulintchenko et al., 2003), which served as a template for the synthesis of DNA import substrates. 2.3 kb – 2.3 kb plasmid sequence from the mitochondrial genome of Zea mays; TIR – terminal inverted repeats of the 11.6 kb plasmid from the mt-genome of Brassica rapa; b – electrophoretic analysis of DNA import substrates (200 ng each) prepared from pCK/GFP/PRmt. 1 – 2732 bp; 2 – 265 bp; 3 – DNA molecular weight marker.

It is known that DNA import into mitochondria occurs through various transport pathways, the efficiency of which depends on the size of the imported molecules (Weber-Lotfi et al., 2015; Tarasenko et al., 2021). Based on this, the role of individual protein components of the mitochondrial membrane in the import of DNA molecules of small (265 bp) and medium (2732 bp) lengths was studied (Fig. 1).

**Fig. 2.**
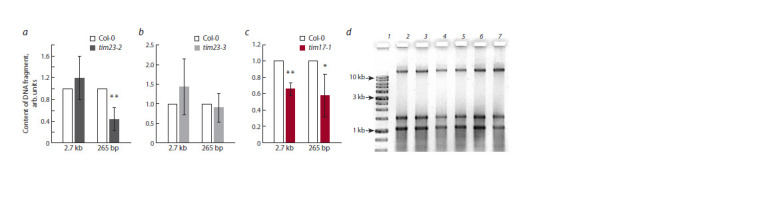
Analysis of exogenous DNA import into isolated Arabidopsis mitochondria DNA fragments 265 bp and 2.7 kb in size were imported into mitochondria of wild-type and mutant Arabidopsis lines: (a) tim23-2, (b) tim23-3, and (c) tim17-1. Mitochondrial DNA extracted from mitochondria after import (d ) was used for qPCR analysis. The amount of the GFP gene fragment (imported DNA) normalized to the content of the NAD4 gene fragment (mtDNA) is shown. The import level in Col-0 is taken as an arbitrary unit. The mean values are shown with standard deviations. * and ** statistically significant differences at p ≤ 0.05 and p ≤ 0.01, respectively. d – mitochondrial nucleic acid preparation used for analysis: 1 – DNA molecular weight marker; 2, 3 – mtDNA from tim23-2; 4, 5 – mtDNA from tim23-3; 6, 7 – mtDNA from tim17-1.

DNA fragments 265 bp and 2.7 kb in size obtained by PCR (see Fig. 1, b) were imported into mitochondria isolated from Arabidopsis plants (in organello), wild-type (Col-0) and knockout mutants for Tim23-2 (tim23-2), Tim23-3 (tim23-3) and Tim17-1 (tim17-1), which are isoforms of key proteins of the Tim17:23 complex. For mitochondria of the tim23-2 mutant, a significant decrease in the import level of a smalllength fragment was shown in comparison with the wild type (Fig. 2, a), while the import level of a medium-sized fragment did not differ significantly. In mitochondria of the tim23-3 mutant, no dependence of both DNA fragments import efficiency on the absence of functional Tim23-3 was found (see Fig. 2, b). Thus, it is obvious that the Tim23-2 protein isoform is part of the DNA import apparatus, performing a specific role in the transfer of DNA molecules, predominantly of short length.

When studying the import into tim17-1 mutant mitochondria, we observed a decrease in the DNA transport efficiency, approximately similar for fragments of both short and medium length (see Fig. 2, c). These results suggest that the Tim17- 1 protein may also be an important participant in the DNA translocation machinery into mitochondria

**Fig. 3.**
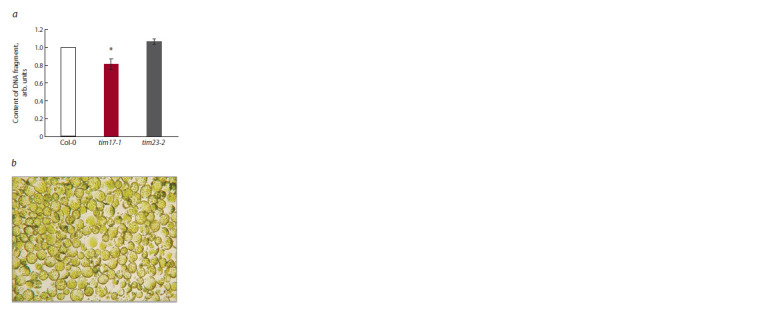
Level of DNA import into mitochondria of protoplasts obtained from Arabidopsis leaves using qPCR. а – 2.7 kb DNA fragment was imported into mitochondria of protoplasts of wild-type and tim17-1 and tim23- 2 knockout lines. After transfection of protoplasts, mitochondria were isolated, followed by mtDNA extraction. The amount of the detected GFP gene fragment normalized to the content of the NAD4 gene fragment is shown. The import level in Col-0 is taken as an arbitrary unit. The mean values are shown with standard deviations. * Statistically significant differences at p ≤ 0.05; b – light microscopy of protoplast integrity after transformation with a DNA fragment and incubation for 20 hours.

In order to verify the data on the import of a DNA fragment of medium length (2.7 kb) into mitochondria isolated from the Tim17 and Tim23 knockout lines (see Fig. 2, a, c), we carried out experiments using Arabidopsis protoplasts obtained from these lines (in vivo) (Fig. 3, a). Protoplasts maintained their integrity for 20 hours (see Fig. 3, b). It was found that the import level of exogenous DNA from the cytoplasm of tim17-1 knockout protoplasts into the mitochondria was indeed reduced, but to a lesser extent than that observed in organello. It can be concluded that the deficiency of mitochondrial Tim17-1 is apparently partially compensated in vivo by certain cellular factors. Compared to tim17-1, the level of mitochondrial import of tim23-2 did not differ from the wild type, similar to what was shown in the isolated organelles (see Fig. 3, a). Taken together, the results obtained in organello using mutant lines lacking the Tim23-2 or Tim17-1 proteins reflect the patterns of DNA transfer in vivo into the mitochondria of protoplasts of these lines.

**Fig. 4.**
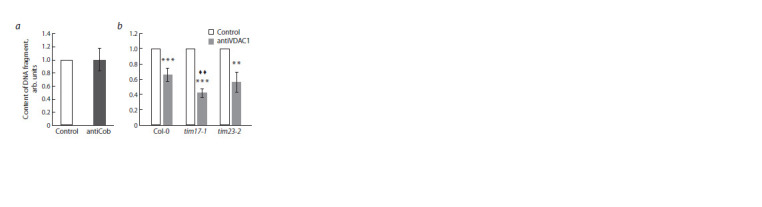
Efficiency of short-length DNA import into isolated Arabidopsis mitochondria in the presence of antibodies to membrane proteins а – determination of possible nonspecific inhibition of transport channels involved in DNA import by antibodies to Cob; b – DNA import efficiency into mitochondria of Col-0 and tim17-1 and tim23-2 knockout lines in the presence of specific antibodies to VDAC1 (antiVDAC1). The amount of the GFP gene fragment normalized to the content of the NAD4 gene fragment is shown. Import level of the 265 bp fragment in Arabidopsis mitochondria without pretreatment with antibodies (control) is taken as an arbitrary unit. The mean values are shown with standard deviations. ** (♦ ♦) and *** – statistically significant differences at p ≤ 0.01 and p ≤ 0.001, respectively; * – differences from control; ♦ – differences from the level of import into Col-0 mitochondria pre-treated with antiVDAC1 (b).

The next task was to investigate the possible relationship of the outer mitochondrial membrane protein VDAC with Tim17-1 and Tim23-2 in the import of short DNA molecules. The use of antibodies to VDAC1 potentially makes it possible to exclude this isoform from participation in the formation of a channel for DNA transfer. It has been shown (Koulintchenko et al., 2003) that the binding of antibodies specific to a certain protein of the outer mitochondrial membrane should inhibit its transport activity. We applied this approach to analyze the efficiency of 265 bp DNA fragments import into isolated wild-type and tim17-1 or tim23-2 mutant mitochondria (Fig. 4).

At first, in order to exclude nonspecific inhibition, we assessed the effect of antibodies that specifically bind mitochondrial apocytochrome b (Cob). This inner membrane protein is the central catalytic subunit of ubiquinol-cytochrome c oxidoreductase (Islas-Osuna et al., 2006). The outer membrane of mitochondria is impermeable to antibodies, so inhibition of transport processes should not occur when using antibodies to Cob. Import level of the 265 bp fragment in wild-type mitochondria after their treatment with antibodies to Cob did not differ from the control sample (see Fig. 4, a). Based on this, experiments were carried out in which DNA fragments 265 bp in size were imported into isolated mitochondria of tim17-1 or tim23-2 mutant lines, pretreated with antibodies to the outer membrane protein VDAC1 (see Fig. 4, b).

According to the data obtained, the level of import of short-length DNA into wild-type mitochondria in the presence of antibodies to VDAC1 was significantly reduced (see Fig. 4, b). It is obvious that this porin isoform is directly involved in the transport of DNA of this length into Arabidopsis mitochondria. Analysis of import into mitochondria lacking functional Tim23-2 showed that in the presence of antibodies to VDAC1, the efficiency of this process did not differ from that in the wild type under the same conditions (see Fig. 4, b). Considering the role of Tim23-2 in the import of short-length DNA (see Fig. 2, a), these results indicate that VDAC1 presumably forms one transport channel with Tim23-2, since inhibition of either component results in an approximately equal decrease in import level. Moreover, the degree of import reduction does not change with simultaneous inactivation of these two transporter proteins located in different mitochondrial membranes (see Fig. 4, b). At the same time, in mitochondria of the tim17-1 line treated with antibodies to VDAC1, we observed an additional decrease in the level of import of the 265 bp fragment (see Fig. 4, b), which indicates the participation of Tim17-1 in the formation of a DNA import channel independent of VDAC1.

It can be suggested that VDAC1 is a companion of Tim23-2, but not Tim17- 1, in the process of translocation of short fragments across the double mitochondrial membrane. At the same time, the Tim17-1 protein, unlike Tim23-2, is involved in the process of import of fragments of both short and medium length, which serves as an additional argument in favor of the independence of Tim17-1 from the channel formed by Tim23-2 and VDAC1

In order to estimate the actual efficiency of import of two DNA fragments into mitochondria of different lines, we calculated the content of imported DNA

in mitochondria in relation to the content of mitochondrial DNA (see the Table). It was shown that the import efficiency of a short fragment is extremely high and amounts to up to 5 % of the amount of mtDNA. Import of the 2.7 kb fragment was much less effective; the amount of DNA penetrated into the organelles was 30 times less than the amount of the imported 265 bp fragment. The data obtained correlate well with known data on the efficiency of import (Koulintchenko et al., 2003), and also provide an additional argument in favor of the existence of separate import pathways for fragments of short and medium length, differing in the intensity of DNA transport through the membrane.

**Table 1.**
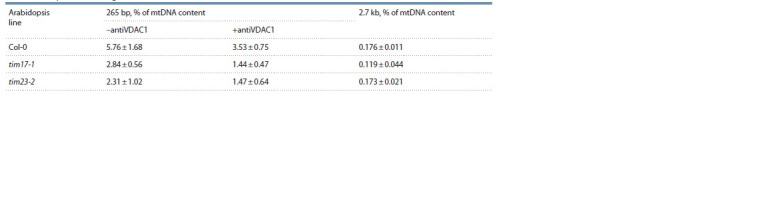
Content of imported DNA fragments in mitochondria relative to mtDNA content

## Discussion

The role of the Tim23 and Tim17 subunits in protein import into mitochondria has been extensively studied (Murcha et al., 2003, 2014; Lister et al., 2004). However, studies of the potential involvement of the TIM17:23 complex or its individual subunits in the DNA import into mitochondria have not been carried out until now. It was previously shown that different isoforms of the Tim17 and Tim23 proteins (Murcha et al., 2007) differ in their ability to complement knockout mutants of orthologous subunits in yeast, suggesting some functional specialization of different isoforms (Murcha et al., 2003). This study focused on the major isoform Tim23-2 and the minor isoform Tim17-1, the role of which in plant mitochondria remains poorly understood. To import foreign DNA fragments of different lengths, we used mitochondria isolated from Arabidopsis Tim23-2 and Tim17-1 knockout lines.

In the in organello system, we showed that both of these proteins are involved in DNA import, with Tim23-2 being more specific with respect to the size of the transferred molecule (see Fig. 2, a). These data were confirmed in the system of DNA import into mitochondria of protoplasts obtained from Arabidopsis knockout lines (Wu et al., 2009). Previously, we developed an effective method for studying the import of DNA fragments into mitochondria following transfection of Arabidopsis protoplasts with these molecules, i. e. under conditions of maintaining the native cellular environment of mitochondria (Tarasenko et al., 2019). This work established a number of regularities, the main one being that the results obtained in protoplasts are consistent with data from studies of DNA import into isolated mitochondria.

The results obtained using protoplasts allow us to conclude that the observed decrease in DNA import into mitochondria lacking Tim17-1 occurs in vivo. Likewise, using protoplasts, it was confirmed that Tim23-2 does not play a role in the import of medium-length DNA. This protein, however, according to in organello experiments, exhibits activity specific for the transfer of short DNA fragments. This property of Tim23-2 is another argument in favor of the existence of several pathways for DNA transfer through the inner membrane, specific to a certain extent with respect to the size of the imported molecules.

Another protein studied, the minor isoform Tim17-1, turned out to be involved in the import of DNA fragments of both short and medium length. The Tim17-1 isoform is characterized by a high level of expression during seed germination; therefore, it is assumed that Tim17-1 may be involved in mitochondrial biogenesis at this stage of development (Wang et al., 2014). The role of this isoform in protein import in the adult Arabidopsis plant is not obvious due to its low level of expression, given an increase in the expression of the other two isoforms throughout plant development. The gradual decrease in the expression level of the Tim17-1 subunit after germination (Wang et al., 2014) may indicate the potential specialization of this isoform in alternative and less important processes.

In addition to the use of knockout mutants, another approach to study the role of mitochondrial membrane proteins in mitochondrial transport processes is the use of specific antibodies to these proteins (Koulintchenko et al., 2003; Murcha et al., 2005). Studies of the VDAC role in DNA import into isolated potato and rat mitochondria were previously carried out using antibodies that recognize the conserved domain of VDAC proteins (Koulintchenko et al., 2003, 2006). However, for any group of organisms, there are no data so far on the role of a specific mitochondrial porin isoform in this process. Previously, we attempted to study the participation of one or another VDAC isoform in DNA import using knockout mutants; however, a decrease in the level of import was not detected in any of these lines, apparently due to compensation for the lack of the protein by other isoforms/transport mechanisms throughout plant development (Tarasenko et al., 2021).

**Fig. 5.**
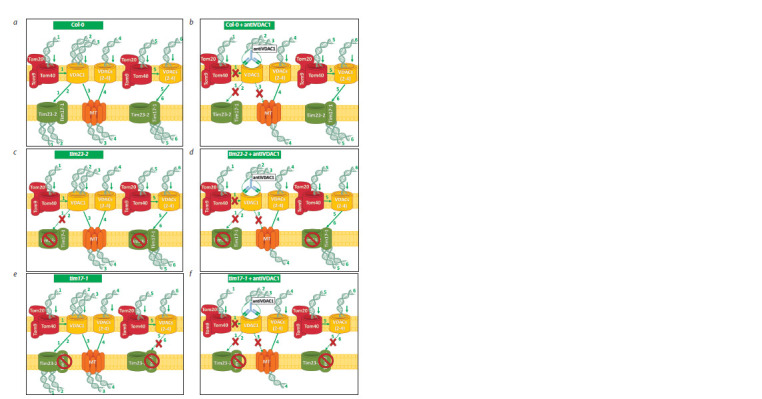
Putative effect of inactivation of mitochondrial membrane proteins on the level of DNA import into mitochondria Inactivation of the transport process was ensured by (a, c, e) using Arabidopsis knockout mutants lacking functional Tim17-1 or Tim23-2 protein, and/or (b, d, f ) inhibition of the VDAC1 protein with specific antibodies. In the case of simultaneous exclusion of VDAC1 and Tim23-2 from the transport process (d ), there was a decrease in the level of import, comparable in intensity to that observed under conditions of inactivation of only one transporter (b or c). With the simultaneous exclusion of VDAC1 and Tim17-1 from the transport process (f ), an additional decrease in efficiency was observed in comparison with the inactivation of one protein (b or e), indicating that these proteins belong to two different channels. 1–6 – pathways for transfer of DNA molecules into the matrix; arrows – direction of DNA transfer; dashed arrows crossed out with a red cross – blocking of the transport route; crossed out circle – absence of a functional protein in the mitochondrial membrane as a result of gene knockout. MT is a complex formed with the participation of mitochondrial transporters.

It is known that the four isoforms of Arabidopsis VDAC, despite a high degree of homology (from 68 to 50 %), apparently have functional specialization (Tateda et al., 2011; Hemono et al., 2020). When assessing protein levels in Arabidopsis mitochondria, the most abundant porin isoform was found to be VDAC1, which has approximately 44,400 copies per mitochondrion (Fuchs et al., 2020). In our work, we used isoform-specific antibodies to Arabidopsis VDAC1 (AT3G01280), which interact with the N-terminus of this protein. It was shown that inhibition of VDAC1 activity by antibodies leads to a decrease in DNA import intensity. This fact suggests that the use of specific antibodies to suppress porin activity has proven to be a more productive approach to studying the role of VDAC isoforms in DNA import than the use of knockout mutants. Thus, we have demonstrated for the first time the involvement of a specific porin isoform, VDAC1, in DNA transfer into mitochondria (Fig. 5, a, b). While a specific function of the VDAC4 isoform has been suggested in the mechanism of tRNA import into Arabidopsis mitochondria (Hemono et al., 2020), this work demonstrates the involvement of the VDAC1 isoform in DNA import. Thus, functional specialization of different VDAC isoforms with respect to the type of nucleic acids (DNA/RNA) and their size cannot be excluded.

We also investigated the possibility of VDAC1 interacting with Tim17-1 or Tim23-2 during DNA translocation. Due to the fact that both Tim17-1 and Tim23-2 are involved in the import of the short 265 bp fragment, we used a DNA substrate of this size. As a result, it was established for the first time that VDAC1 apparently forms a common transport channel with Tim23-2. We observed an equal decrease in the level of shortlength DNA import upon inhibition of one of these proteins and the absence of an additional import decrease upon their simultaneous inactivation (see Fig. 5, c, d ).

An additional DNA import decrease observed in the tim17-1 knockout mitochondria upon inhibition of the VDAC1 protein with antibodies allows us to conclude that the Tim17-1 and VDAC1 proteins apparently belong to two independent transport channels (see Fig. 5, e, f ). We hypothesize that the outer membrane companion protein for Tim17-1 might be one of the other porin isoforms (see Fig. 5, a, e). The absence of complete inhibition of DNA import into Arabidopsis mitochondria upon inactivation of any of the studied membrane proteins is consistent with the hypothesis that these organelles have multiple DNA transfer pathways (Weber-Lotfi et al., 2015; Tarasenko et al., 2021).

The extent to which other VDAC isoforms and Tim17:23 components participate in DNA import, whether there is specificity for them in terms of the size of the transferred molecule, and the nature of the relationship between the VDAC isoforms and the Tim17-2, Tim17-3, Tim23-1, Tim23-3 proteins remains to be determined in further studies

## Conclusion

The study of the role of the TIM17:23 complex proteins of the inner mitochondrial membrane in the DNA import mechanism made it possible to establish their participation in this process, as well as to reveal the possibility of their joint functioning with VDAC1. Given that the specificity of DNA import with respect to the size of the transferred molecule is likely determined at the level of the inner membrane, our data have deepened our understanding of the import mechanism and expanded the possibilities for developing a system for transforming the mitochondrial genome.

## Conflict of interest

The authors declare no conflict of interest.
